# Role of Glutathione in Buffering Excess Intracellular Copper in *Streptococcus pyogenes*

**DOI:** 10.1128/mBio.02804-20

**Published:** 2020-12-01

**Authors:** Louisa J. Stewart, Cheryl-lynn Y. Ong, May M. Zhang, Stephan Brouwer, Liam McIntyre, Mark R. Davies, Mark J. Walker, Alastair G. McEwan, Kevin J. Waldron, Karrera Y. Djoko

**Affiliations:** a Department of Biosciences, Durham University, Durham, United Kingdom; b School of Chemistry and Molecular Biosciences and Australian Infectious Diseases Research Centre, The University of Queensland, St. Lucia, Australia; c Department of Microbiology and Immunology, University of Melbourne, at the Peter Doherty Institute for Infection and Immunity, Melbourne, Victoria, Australia; d Biosciences Institute, Faculty of Medical Sciences, Framlington Place, Newcastle University, Newcastle upon Tyne, United Kingdom; University of Arizona

**Keywords:** copper homeostasis, copper export, metal buffer, glutathione, group A *Streptococcus*, copper stress, copper tolerance

## Abstract

Copper (Cu) is an essential metal for bacterial physiology but in excess it is bacteriotoxic. To limit Cu levels in the cytoplasm, most bacteria possess a transcriptionally responsive system for Cu export. In the Gram-positive human pathogen Streptococcus pyogenes (group A *Streptococcus* [GAS]), this system is encoded by the *copYAZ* operon. This study demonstrates that although the site of GAS infection represents a Cu-rich environment, inactivation of the *copA* Cu efflux gene does not reduce virulence in a mouse model of invasive disease. *In vitro*, Cu treatment leads to multiple observable phenotypes, including defects in growth and viability, decreased fermentation, inhibition of glyceraldehyde-3-phosphate dehydrogenase (GapA) activity, and misregulation of metal homeostasis, likely as a consequence of mismetalation of noncognate metal-binding sites by Cu. Surprisingly, the onset of these effects is delayed by ∼4 h even though expression of *copZ* is upregulated immediately upon exposure to Cu. Further biochemical investigations show that the onset of all phenotypes coincides with depletion of intracellular glutathione (GSH). Supplementation with extracellular GSH replenishes the intracellular pool of this thiol and suppresses all the observable effects of Cu treatment. These results indicate that GSH buffers excess intracellular Cu when the transcriptionally responsive Cu export system is overwhelmed. Thus, while the *copYAZ* operon is responsible for Cu *homeostasis*, GSH has a role in Cu *tolerance* and allows bacteria to maintain metabolism even in the presence of an excess of this metal ion.

## Abstract (precis)

The control of intracellular metal availability is fundamental to bacterial physiology. In the case of copper (Cu), it has been established that rising intracellular Cu levels eventually fill the metal-sensing site of the endogenous Cu-sensing transcriptional regulator, which in turn induces transcription of a copper export pump. This response caps intracellular Cu availability below a well-defined threshold and prevents Cu toxicity. Glutathione, abundant in many bacteria, is known to bind Cu and has long been assumed to contribute to bacterial Cu handling. However, there is some ambiguity since neither its biosynthesis nor uptake is Cu-regulated. Furthermore, there is little experimental support for this physiological role of glutathione beyond measuring growth of glutathione-deficient mutants in the presence of Cu. Our work with group A *Streptococcus* provides new evidence that glutathione increases the threshold of intracellular Cu availability that can be tolerated by bacteria and thus advances fundamental understanding of bacterial Cu handling.

## INTRODUCTION

Bacteria have been exposed to environmental copper (Cu) since the Great Oxidation Event, when the rise in atmospheric O_2_ levels led to its solubilization from minerals. There is also evidence that recent evolution of plant, animal, and human pathogens has been influenced by the anthropogenic release of Cu into soils, for instance via mining activities and the legacy of using Cu salts and compounds in industrial-scale biocides (1). In addition, bacteria encounter elevated levels of Cu in microenvironments within a eukaryotic host. Bacterial predation induces an increase in intracellular Cu levels in protozoa (2), while phagocytosis stimulates uptake and accumulation of Cu in murine macrophages (3, 4). Studies of more complex animal models of infectious disease and human infections further suggest that infection triggers systemic changes in host Cu levels and that the specific sites of inflammation are usually, though not always, Cu-rich (5–9). The prevailing model is that Cu exerts a direct antibacterial action and/or supports the antibacterial function of innate immune cells (10).

Cu can be bacteriotoxic because it is a thermodynamically competitive metal for protein binding (11). Extracellular Cu invariably enters the bacterial cytoplasm via uptake processes that remain poorly understood. Once inside, Cu fills the available Cu-binding sites in proteins and other biomolecules, beginning with the tightest affinity and eventually associating with the weakest affinity sites. Within this hierarchy of binding sites are the allosteric sites in Cu-sensing transcriptional regulators, which, when metalated by Cu, activate expression of a Cu efflux pump (12). In undertaking this role, the Cu sensor and export pump together impose an upper threshold of Cu availability in the cytoplasm. This system ensures that only native, stable, high-affinity Cu sites are metalated by Cu, and at the same time, prevents adventitious, nonspecific, noncognate, weaker-binding sites from becoming mismetalated. Such mismetalation events can inactivate key enzymes and, consequently, impair bacterial growth and viability (13–16).

Additional cytoplasmic components are thought to limit Cu availability by chelating or “buffering” this metal ion. These components include bacterial metallothioneins (17), Cu storage proteins (18), and Cu-binding metallochaperones (19, 20), which are often, though not always, transcriptionally regulated by the endogenous Cu sensors. Mutant bacterial strains lacking these proteins typically display a Cu-sensitive growth phenotype. Non-protein components, particularly the low-molecular-weight thiol glutathione (GSH), are also assumed to buffer Cu (21), although their uptake or biosynthesis is not transcriptionally induced in response to Cu treatment (15, 20, 22, 23). Addition of GSH protects purified metalloenzymes from inactivation by Cu (13). Growth of bacterial mutant strains that are impaired in GSH uptake (24) or biosynthesis (25–27) are all inhibited by added Cu, especially if the Cu efflux pump (25, 26) or Cu-binding metallochaperone (27) in the organism is also inactivated.

Beyond growth analysis, there is currently little experimental support for a physiological role of GSH in buffering Cu in bacteria. Perhaps the clearest, albeit indirect, line of evidence was obtained using a Δ*gshB* Δ*atx1* mutant of *Synechocystis* lacking the GSH biosynthesis enzyme GshB and the cytoplasmic Cu-binding metallochaperone Atx1. This mutant failed to repress expression of Zn-regulated genes in response to elevated Zn (27). *In vitro* metal- and DNA-binding experiments (28) suggest that the absence of GSH and the metallochaperone leads to an increase in intracellular Cu availability, which mismetalates the allosteric site of the Zn sensor Zur and thus interferes with Zn sensing.

Like most bacteria, the Gram-positive human pathogen Streptococcus pyogenes (group A *Streptococcus* [GAS]) possesses a system for Cu sensing and efflux, which is encoded by the *copYAZ* operon (29). In this work, we examine whether *copA*, encoding the Cu-effluxing P_1B-1_-type ATPase, plays a critical role in GAS pathogenesis, as demonstrated for other bacterial pathogens (7, 30–32). We show that GAS occupies a Cu-rich environment during infection of a mouse model of invasive disease, and yet inactivation of *copA* does not significantly reduce GAS virulence. This unexpected observation leads us to investigate the effects of Cu treatment on the cellular biochemistry and physiology of GAS. The results provide key insights into the importance of GSH in cytoplasmic Cu buffering to supplement the transcriptionally responsive Cu sensing and efflux system. This additional buffering extends the range of intracellular Cu concentrations that can be tolerated by bacteria and thus prevents a sudden or abrupt transition from Cu homeostasis to Cu stress upon exposure to an excess of this metal ion.

## RESULTS

### Initial characterization of a Δ*copA* mutant.

The *copYAZ* operon in GAS has been previously shown to resemble other Cop systems in Gram-positive bacteria (29) (see Fig. S1A in the supplemental material). Consistent with a role in Cu efflux, expression of this operon functionally complemented a heterologous Escherichia coli Δ*copA* mutant strain (29). *In silico* analyses found one additional open reading frame downstream of *copZ* (see Fig. S1A). It encodes a small, uncharacterized protein (56 amino acids) with an N-terminal transmembrane domain, a putative metal-binding C-X_3_-M-H motif at the C terminus, and no characterized homologue. This gene is absent from *copYAZ* operons in other Gram-positive bacteria and its function in Cu homeostasis is unknown.

10.1128/mBio.02804-20.3 FIG S1 Initial characterization of GAS Δ*copA* mutant strain. (A) Working model of the Cop system. Structural models were generated in SWISS-MODEL (A. Waterhouse, M. Bertoni, S. Bienert, G. Studer, et al., Nucleic Acids Res 46:W296–W303, 2018) using the following PDB IDs as the templates: CopA (3j09), CopZ (1k0v), CopY (1fwz). Based on the *E. hirae* and S. pneumoniae paradigm, GAS CopY would bind Zn in its repressor form. Exchange of Zn with Cu during conditions of Cu excess would promote dissociation of CopY from its DNA binding site (*cop* box, ATTTACAAATGTAGTT) (H. Glauninger, Y. Zhang, K. A. Higgins, A. D. Jacobs, et al., Chem Sci 9:105–118, 2018; H. O’Brien, J. W. Alvin, S. V. Menghani, Y. Sanchez-Rosario, et al., mSphere 5:e00411-20, 2020, https://doi.org/10.1128/mSphere.00411-20; D. Strausak and M. Solioz, J Biol Chem 272:8932–8936, 1997). CopZ functions presumably as a cytoplasmic Cu-binding metallochaperone. CopA is a P_1B-1_-type ATPase that extrudes excess Cu from the cytoplasm to the extracellular space. CopX has no known homologue outside GAS and has not been characterized. Organization of the *copYAZX* operon is shown at the bottom. Gene map was generated using the genome sequence of GAS MGAS5005 (NCBI GenBank accession no. CP000017.2, region *M5005_Spy_1406* to *M5005_Spy_1403*). (B) (i) Basal and (ii) Cu-dependent transcription profiles of *cop* genes. GAS was cultured with 0 μM (for panel i; *n* = 3) or 5 μM (for panel ii; *n* = 3) of added Cu. Bacteria were harvested at *t* = 4 h. Amounts of transcripts were quantified by qPCR and normalized to *holB* (for panel i) or to the corresponding untreated samples (for panel ii). nd, not detectable over background or nonspecific amplification. (C) Growth profiles. GAS was cultured with added Cu as indicated (*n* = 3). Final OD_600_ values were measured at *t* = 8 h. (D) Levels of intracellular Cu. GAS was cultured with added Cu as indicated (*n* = 3). Bacteria were harvested at *t* = 4 h and intracellular Cu levels were determined by ICP-MS. All figures show GAS wild-type (WT, black), Δ*copA* mutant (red), and *copA^+^* complemented mutant (blue) strains. Download FIG S1, TIF file, 0.8 MB. Copyright © 2020 Stewart et al. 2020 Stewart et al. This content is distributed under the terms of the Creative Commons Attribution 4.0 International license.

For the present study, a non-polar Δ*copA* mutant of GAS M1T1 strain 5448 was constructed. This mutation did not alter basal expression of downstream *cop* genes (see Fig. S1Bi). As anticipated, the Δ*copA* mutant was more susceptible to growth inhibition by added Cu than was the wild type (see Fig. S1C). This mutant also accumulated more intracellular Cu (see Fig. S1D), leading to increased expression of the other *cop* genes compared to the wild type (see Fig. S1Bii). Marker rescue (*copA^+^*) restored the expression of both *copA* and wild-type phenotypes (see Fig. S1B to D).

### Deletion of *copA* does not lead to a loss of virulence in a mouse model of infection.

**FIG 1:**
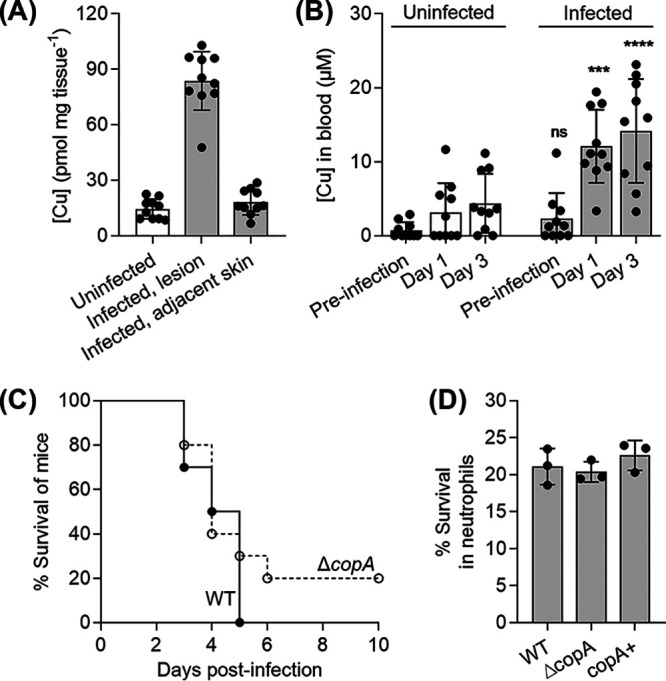
Changes in Cu levels during GAS infection and the effect of a *copA* mutation on GAS virulence in host infection models. (A) Cu levels in mouse lesions. Mice were infected subcutaneously with GAS wild-type strain or left uninfected (*n* = 10 each). After 3 days, skin from uninfected mice, and both skin lesions and healthy skin adjacent to the lesions from infected mice were excised. Total Cu levels were measured by ICP-MS and normalized to the weight of the tissues. Cu levels in infected lesions were higher than those in adjacent healthy skin (*P* < 0.0001) or skin from uninfected mice (*P* < 0.0001). (B) Cu levels in mouse blood. Mice were infected subcutaneously with GAS wild-type strain or left uninfected (*n* = 10 each). Blood was collected and total Cu levels were measured by ICP-MS. Values below the detection limit were represented as zero. Cu levels in the blood of infected mice on days 1 and 3 were higher from those in the blood of uninfected mice (***, *P* = 0.0001; ****, *P* < 0.0001). ns, *P* = 0.81 (versus uninfected mice). (C) Virulence in an *in vivo* mouse model of infection. Mice were infected subcutaneously with GAS wild-type (WT) or Δ*copA* mutant strains (*n* = 10 each). The number of surviving mice was counted daily up to 10 days post-infection. Differences in survival curves were analyzed using the Mann-Whitney test, which found no statistical difference (*P* = 0.099). (D) Virulence in an *ex vivo* human neutrophil model of infection. Human neutrophils were infected with GAS wild-type (WT), Δ*copA*, or *copA^+^* strains (*n* = 3 each). Survival of bacteria relative to the input was measured after 0.5 h. There was no difference between survival of the Δ*copA* mutant compared with the WT (*P* = 0.87) or *copA*^+^ (*P* = 0.35) strains.

To determine whether the Cop system and its interactions with host Cu have an effect on GAS pathogenesis, an established invasive disease model using transgenic human-plasminogenized mice was used (33). Mice subcutaneously infected with wild-type GAS developed ulcerative skin lesions at the site of injection after 1 day. These lesions were excised 3 days post-infection and were found to contain more Cu than adjacent healthy skin or skin from uninfected mice (Fig. 1A). Consistent with these results, the *copYAZ* operon was upregulated in GAS isolated from infected mouse tissues compared to those grown in THY medium (34). There was also an increase in Cu levels in mouse blood after 3 days of infection (Fig. 1B). Notably, these Cu levels in the blood are comparable to those measured in the sera of mice infected with the fungal pathogen Candida albicans or the parasite Plasmodium berghei (5). These observations support a model where redistribution of host Cu is a feature of the general immune response to infection (5).

Comparing the survival of mice post-infection, no statistically significant difference was observed whether mice were infected with the wild type or the *ΔcopA* mutant (*P* = 0.0991; Fig. 1C). Although no single animal model can fully represent the complex features of human streptococcal diseases (35), consistent with *in vivo* findings, the Δ*copA* mutant was no more susceptible to killing by human neutrophils compared with the wild-type or *copA*^+^ mutant strains in an *ex vivo* infection assay (Fig. 1D). In addition, recent reports did *not* identify the *cop* genes to be fitness determinants during *ex vivo* infection of human blood (36) or *in vivo* soft tissue infection in mice (37). These results imply that, despite the systemic and niche-specific elevated levels of host Cu, the Cu efflux pump CopA is not essential for GAS virulence in this model.

### Cu treatment leads to defects in the late exponential phase of growth.

**FIG 2:**
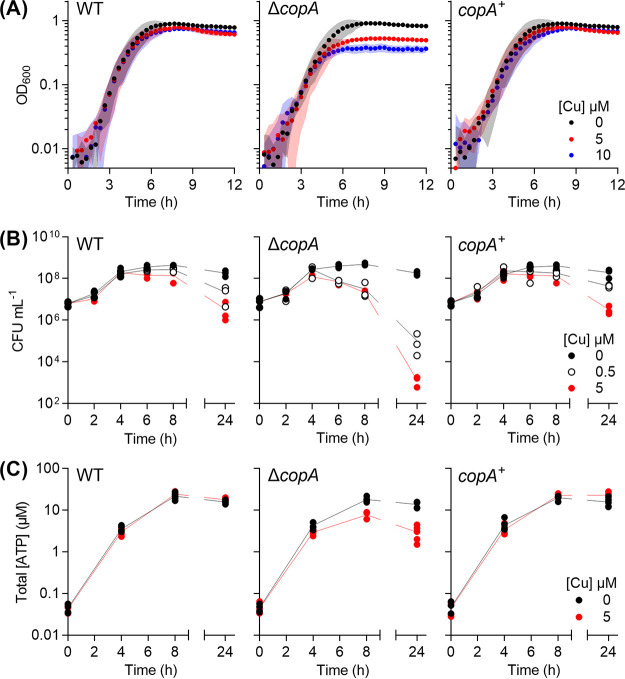
Cu-dependent defects in growth and viability. GAS strains were cultured with added Cu as indicated. (A) Growth. Cultures (*n* = 3) were grown in microtiter plates and OD_600_ values were recorded every 20 min. Cu treatment suppressed growth of Δ*copA* cultures (*P* = 0.034 for 5 μM Cu, *P* < 0.0001 for 10 μM Cu). (B) Plating efficiency. Cultures (*n* = 3) were plated out at the indicated time points and the number of CFU was enumerated. Cu treatment suppressed plating efficiency of the Δ*copA* cultures (*P* < 0.0001 for both 0.5 and 5 μM Cu). (C) Total ATP levels. Cultures (*n* = 5) were sampled at the indicated time points and total ATP levels were determined. Cu treatment suppressed ATP production in the Δ*copA* cultures (*P* < 0.0001). All statistical analyses were versus 0 μM Cu.

The lack of a virulence defect for the *ΔcopA* mutant *in vivo* prompted us to examine the impact of Cu treatment on GAS physiology *in vitro*. Addition of Cu (up to 10 μM) to the culture medium did not affect the doubling time of the Δ*copA* mutant during the exponential phase of growth, but it did reduce the final culture yield (Fig. 2A; see Fig. S2A and B). This phenotype was reproduced during growth in the presence of glucose or alternative carbon sources (see Fig. S2C). Under each condition, growth of Cu-treated cultures ceased upon reaching approximately the same optical density at 600 nm (OD_600_; ∼0.35) regardless of growth rate, indicating that the growth defect was related to bacterial cell numbers and/or growth stage. Consistent with this interpretation, Cu treatment did not affect growth in the presence of mannose (see Fig. S2C) or limiting amounts of glucose (see Fig. S2D), since neither experimental condition supported growth of GAS beyond an OD_600_ of ∼0.35.

10.1128/mBio.02804-20.4 FIG S2 Cu-dependent defects in growth. (A) Effects on exponential doubling time (i) and final culture density (ii). GAS Δ*copA* mutant strain was cultured in microtiter plates with added Cu as indicated (*n* = 6). Doubling times were calculated by linear regression of log_2_(OD_600_) values between *t* ∼ 1 h and *t* ∼ 4 h. Final OD_600_ values were determined at *t* = 8 h. ****, *P* < 0.0001 (versus untreated culture). (B) Growth in bulk liquid cultures. This experiment was performed to confirm that the growth defects observed in Fig. 2A were not an artifact of bacterial culture in microtiter plates. GAS Δ*copA* mutant strain was cultured in 40 ml of growth medium in 50-ml tubes, with added Cu salts as indicated (*n* = 2). OD_600_ values were recorded every 2 h. (C) Effect of carbon sources. GAS Δ*copA* mutant strain was cultured in microtiter plates with 0.5% (wt/vol) each of glucosamine (GlcN), *N-*acetylglucosamine (GlcNac), fructose (Fru), or mannose (Man) as the sole carbon source, with added Cu as indicated (*n* = 3). (D) Effect of glucose availability. GAS Δ*copA* mutant strain was cultured in microtiter plates with various concentrations of glucose (% [wt/vol]) as the sole carbon source, with added Cu as indicated (*n* = 3). Download FIG S2, TIF file, 1.0 MB. Copyright © 2020 Stewart et al. 2020 Stewart et al. This content is distributed under the terms of the Creative Commons Attribution 4.0 International license.

Parallel assessments of plating efficiency and total ATP levels confirmed that differences between Cu-treated and untreated cultures appeared only in the late exponential or early stationary phase of growth (after ∼4 h when grown in the presence of glucose; Fig. 2B and C). There were clear decreases in the plating efficiency and ATP production by Cu-treated Δ*copA* cultures during this period compared to the untreated control.

### Cu treatment leads to metabolic arrest in the late exponential phase of growth.

GAS is a lactic acid bacterium. Under our experimental conditions, this organism carried out homolactic fermentation and generated lactic acid as the major end product (see Fig. S3A and B). However, Cu-treated Δ*copA* cultures did not acidify the growth medium (see Fig. S3C), leading us to hypothesize that Cu treatment impairs fermentation in GAS.

10.1128/mBio.02804-20.5 FIG S3 Cu-dependent effects on lactic acid fermentation. (A) Fermentation pathways in GAS. (B) Levels of fermentation end products after 24 h of growth. GAS was cultured with added Cu as indicated for t = 24 h (*n* = 3). Amounts of lactate (i), glucose (ii), pyruvate (iii), and acetate (iv) in the extracellular spent medium were determined. The results confirm that GAS carried out homolactic fermentation under our experimental conditions. Untreated cultures consumed 22.6 ± 0.5 mM of glucose and secreted 36 ± 2 mM of lactic acid (ca. 80% of the carbon yield), along with small amounts of pyruvate (3.1 ± 0.6 mM, ca. 7%) and acetate (0.7 ± 0.1 mM, ca. 1.5%). Cu treatment strongly suppressed lactate production (*P* < 0.0001) and glucose consumption (*P* < 0.0001). Cu treatment did not clearly affect pyruvate production (ns, *P* = 0.64), but it did have a small inhibitory effect on acetate production (**, *P* = 0.002). (C) Medium acidification. Phenol red was present in our culture medium as a pH indicator. GAS was cultured with added Cu as indicated. After 24 h, the microtiter plates were centrifuged, and the culture supernatants were transferred to a fresh microtiter plate and photographed. One representative photograph from numerous independent experiments is shown. (D) Time-dependent production of fermentation end products. GAS was cultured with added Cu as indicated (*n* = 3). Cultures were sampled at the indicated time points and amounts of glucose (i) and pyruvate (ii) in the extracellular spent medium were determined. Cu treatment clearly affected glucose consumption in the Δ*copA* mutant (*P* < 0.0001). (E) Western blot analysis of GapA in cell extracts. GAS was cultured without (–) or with (+) 5 μM of added Cu for *t* = 4 h. Bacteria were harvested and lysed by bead-beating. Exactly 5 μg of lysates were loaded and resolved on a 10% SDS-PAGE gel using Tris-glycine running buffer. The gel was electroblotted onto a polyvinylidene difluoride membrane. Rabbit α-GapA (1:1,000; A. J. Walker and M. J. Walker, unpublished data) was used for immunoblotting and detection of GapA (GAPDH). Rabbit α-IgG-HRP conjugate (1:1,000; Sigma) was used as the secondary antibody. The membrane was developed with 3,3′-diaminobenzidine and hydrogen peroxide. Purified recombinant GapA (A. J. Walker and M. J. Walker, unpublished data) was used as a positive control. A representative blot from three independent experiments is shown. Download FIG S3, TIF file, 1.5 MB. Copyright © 2020 Stewart et al. 2020 Stewart et al. This content is distributed under the terms of the Creative Commons Attribution 4.0 International license.

**FIG 3:**
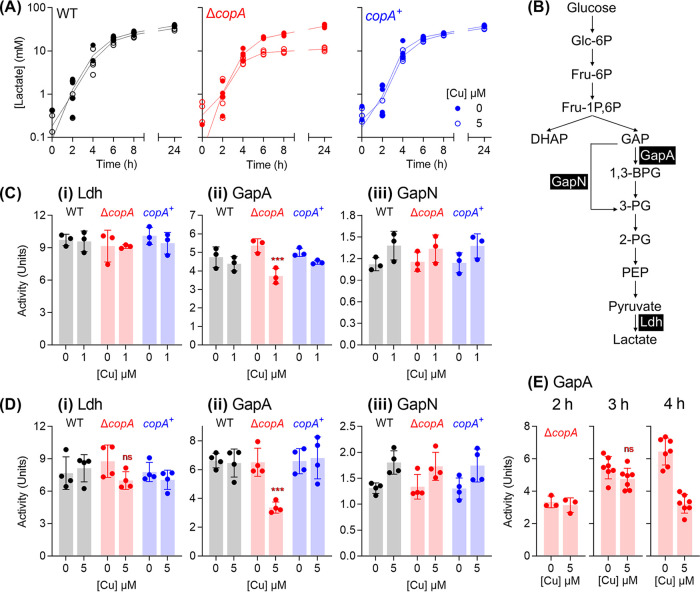
Cu-dependent defects in glycolysis and homolactic fermentation. (A) Lactate production. GAS strains were cultured with added Cu as indicated (*n* = 3). Amounts of lactate secreted to the extracellular culture medium were measured at the indicated time points. Cu treatment suppressed lactate production in the Δ*copA* cultures (*P* < 0.0001). (B) Fermentation pathway in GAS. Enzymes of interest, namely, GapA (NAD^+^-dependent GAPDH, M5005_SPy_0233), GapN (NADP^+^-dependent GAPDH, M5005_SPy_1119), and Ldh (lactate dehydrogenase, M5005_SPy_0873) are shown. (C and D) Activity of glycolytic enzymes Ldh (i), GapA (ii), and GapN (iii). GAS strains were cultured for *t* = 4 h with 0 or 1 μM added Cu (*n* = 3) (C) or 0 or 5 μM added Cu (*n* = 4) (D). Enzyme activities were determined in cell extracts. Cu treatment decreased GapA activity in Δ*copA* cultures (***, *P* = 0.0004). ns, *P* = 0.14. (E) GapA activity over time. GAS Δ*copA* mutant strain was cultured with added Cu as indicated for *t* = 2 h (*n* = 3), 3 h (*n* = 7), or 4 h (*n* = 7). Enzyme activities were determined in cell extracts. Cu treatment did not have an effect on GapA activity at *t* = 2 h (*P* = 0.99) or 3 h (ns, *P* = 0.18), but it strongly inhibited GapA activity at *t* = 4 h (*P* < 0.0001). All statistical analyses were versus 0 μM Cu.

Consistent with this proposal, Cu-treated Δ*copA* cultures produced ∼50% less lactic acid and consumed ∼50% less glucose compared to the untreated control (Fig. 3A; see also Fig. S3Bi and ii). Pyruvate production remained unchanged (see Fig. S3Biii). There is no evidence of a shift toward mixed-acid fermentation since the reduction in lactate levels was not accompanied by a concomitant increase in acetate levels (see Fig. 3Biv). Ethanol levels were undetectable (detection limit, ∼0.2 mM).

Differences in lactate production between Cu-treated and untreated Δ*copA* cultures appeared, again, only after ∼4 h of growth (Fig. 3A). While our methods are not sufficiently sensitive to detect small changes in glucose levels at earlier time points, it is clear that Cu-treated Δ*copA* cultures did not consume glucose beyond *t* ∼ 4 h (see Fig. S3Di). Pyruvate production was, again, not affected at any time point (see Fig. S3Dii). These results suggest that Cu treatment leads to defects in metabolism but only after entry into the late exponential phase of growth.

### Cu treatment results in a loss of GapA activity in the late exponential phase of growth.

The loss of lactate production, but not pyruvate, implies that lactate dehydrogenase (Ldh) is inactivated (Fig. 3B). To test this proposal, we cultured GAS in the absence or presence of added Cu for 4 h, prepared whole-cell extracts, and measured Ldh activity. Figure 3Ci and 3Di show that Ldh remained active in all strains, regardless of Cu treatment.

What, then, is the target of Cu intoxication in GAS? This bacterium does not possess a tricarboxylic acid cycle or the biosynthesis pathways for multiple amino acids, vitamins, and cofactors (e.g., heme). Thus, it lacks obvious candidate iron-sulfur cluster enzymes that are destabilized by excess Cu ions in other systems (13). In an attempt to develop a molecular explanation for the loss of fermentation, the activity of the two GAPDH (glyceraldehyde-3-phosphate dehydrogenase) enzymes in GAS, namely, the classical, phosphorylating, ATP-generating GapA and the alternative, nonphosphorylating GapN, was examined (Fig. 3B). GapA has been identified as a target of Ag and Cu poisoning in E. coli (38) and Staphylococcus aureus (39), respectively, and as such, it is a likely candidate for Cu poisoning in GAS. As expected, Cu treatment led to a decrease in GapA activity in Δ*copA* mutant cells (Fig. 3Cii and Dii), which would explain the reduction in lactate secretion (Fig. 3A) and ATP production (Fig. 2C). The reduction in GapA activity would also cause upstream glycolytic precursors to accumulate, with consequent feedback inhibition of downstream enzymes (40), as well as glucose phosphorylation and uptake (see Fig. S3Bii and S3Di) (41, 42).

This Cu-dependent inhibition is specific to GapA since there was no reduction in GapN activity (Fig. 3Ciii and Diii). Given that there was no detectable change in GapA protein levels in cell extracts (see Fig. S3E), these observations are consistent with mismetalation of GapA, as established recently for the GapA homologue in S. aureus (39). The excess Cu ions likely bind to the conserved Cys and His residues at the catalytic site, as suggested previously for the binding of Ag ions to GapA from E. coli (38).

Remarkably, when cultures were sampled earlier (at *t* = 2 and 3 h), no difference was observed between GapA activity in Cu-treated and control Δ*copA* cells (Fig. 3E). The timing of GapA inhibition, i.e., at the onset of the late exponential phase of growth (at *t* = 4 h; Fig. 3E), coincided with the arrest in bacterial growth and metabolism, supporting the hypothesis that GapA is a key target of Cu intoxication in GAS.

### Cu treatment leads to misregulation of metal homeostasis in late exponential phase of growth.

**FIG 4:**
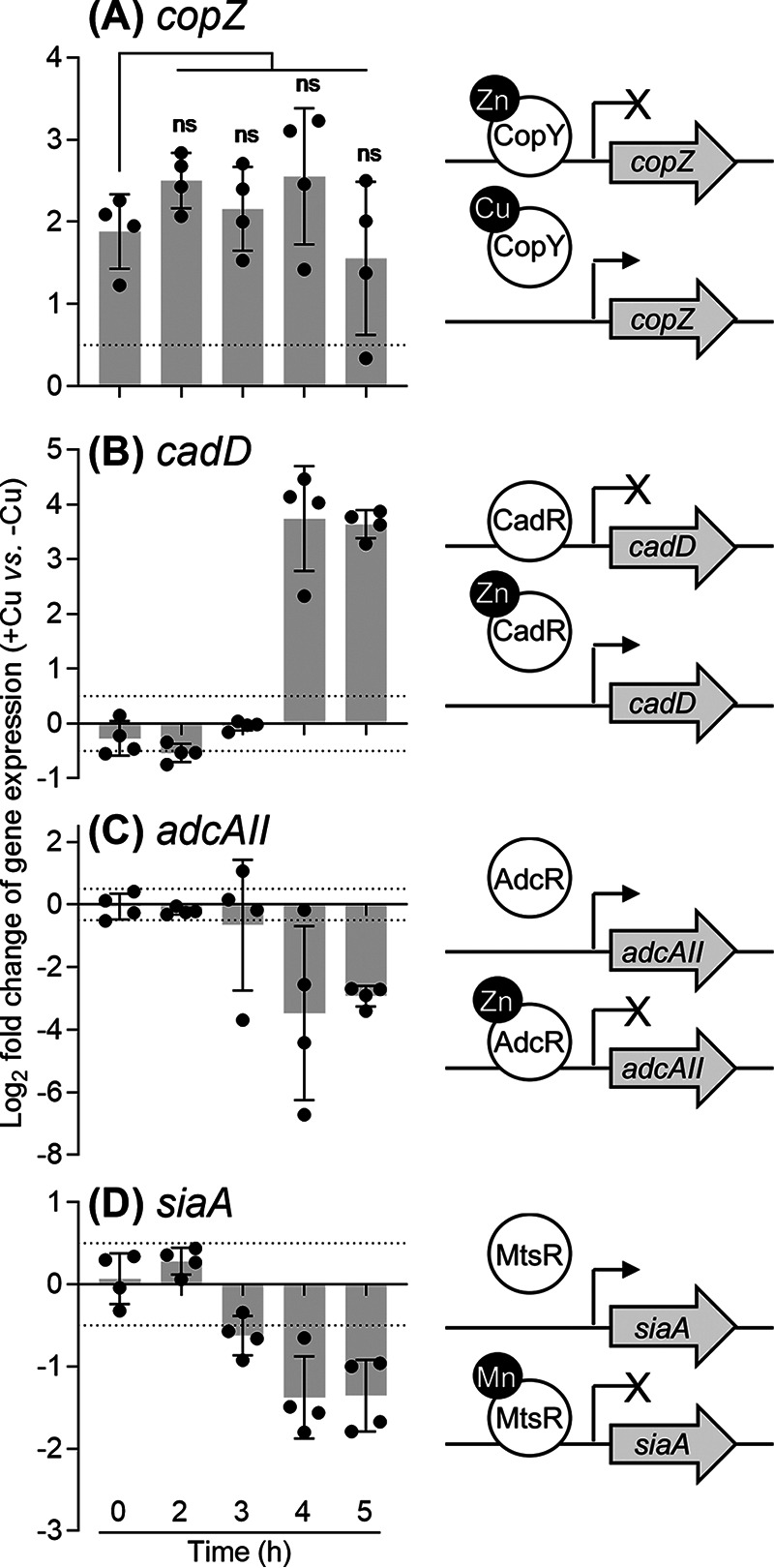
Cu-dependent misregulation of metal homeostasis genes. GAS Δ*copA* mutant strain was cultured with or without added 0.5 μM Cu for the indicated times (*n* = 4). Transcript levels in Cu-treated cultures were determined by qPCR and normalized to the corresponding untreated samples that were cultured for the same time periods. Dotted horizontal lines represent the limit of the assay (log_2_FC = ±0.5). A schematic representation of each gene and its cognate metallosensor is shown. Transcription of *copZ* or *cadD* is derepressed upon binding of Cu to CopY or Zn (or Cd) to CadR, respectively. Transcription of *adcAII* or *siaA* is repressed upon binding of Zn to AdcR or Mn (or Fe) to MtsR, respectively. (A) *copZ*. Cu treatment induced *copZ* expression *t* = 0 h (*P* = 0.0037 versus log_2_FC = 0). This magnitude of induction remained unchanged over the growth period (ns, *P* = 0.53, 0.94, 0.47, and 0.90 for *t* = 2, 3, 4, and 5 h, respectively, versus *t* = 0 h). (B) *cadD*. Cu treatment upregulated *cadD* expression at *t* = 4 and 5 h (*P* = 0.0044 and < 0.0001, respectively, versus log_2_FC = 0). (C) *adcAII*. Cu treatment downregulated *adcAII* expression at *t* = 4 and 5 h (*P* = 0.035 and 0.0004, respectively, versus log_2_FC = 0). (D) *siaA*. Cu treatment downregulated *siaA* expression at *t* = 3, 4, and 5 h (*P* = 0.014, 0.012, and 0.084, respectively, versus log_2_FC = 0).

The puzzling but consistent, 4-h delay in the onset of all observable phenotypes led us to hypothesize that there was a time-dependent shift in Cu handling by GAS. To test this proposal, the response of the Cu sensor CopY was measured by monitoring expression of *copZ* during growth in the presence of the lowest inhibitory concentration of added Cu (0.5 μM; see Fig. S2Aii). The results show that *copZ* transcription was upregulated ∼4-fold immediately upon Cu exposure (*t* = 0 h, in which ∼12 min passed between the addition of Cu into the culture, centrifugation, and the addition of lysis buffer; Fig. 4A). This level of upregulation remained largely unchanged during growth (measured up to 5 h; Fig. 4A), even though intracellular Cu levels continued to rise (see Fig. S4). These results suggest that the CopY sensor became fully metalated and expression of *copZ* reached its maximum at *t* = 0 h post-challenge with added Cu. These data also establish that the *copYAZ* operon is transcriptionally induced before the onset of observable growth defects (hereafter referred to as Cu “stress”).

10.1128/mBio.02804-20.6 FIG S4 Time-dependent changes in intracellular Cu levels. GAS Δ*copA* mutant strain was cultured with 0 μM (black) or 0.5 μM (red) of added Cu (*n* = 3). Cultures were sampled at the indicated time points, plated out for colony counting, and subjected to metal analyses by ICP-MS. Total levels of intracellular Mn, Fe, Zn, and Cu are presented as numbers of metal atoms per cell. Download FIG S4, TIF file, 0.3 MB. Copyright © 2020 Stewart et al. 2020 Stewart et al. This content is distributed under the terms of the Creative Commons Attribution 4.0 International license.

We concurrently measured the expression of genes that are controlled by other metalloregulators, namely *adcAII* (regulated by AdcR, a MarR-family Zn-sensing transcriptional corepressor [43]), *siaA* (controlled by MtsR, a DtxR-family Mn/Fe-sensing corepressor [44]), and *cadD* (regulated by CadC, an ArsR-family Zn/Cd-sensing derepressor [45]). Clear changes in the expression levels of all three genes were detected in response to Cu treatment. While *adcAII* and *siaA* were downregulated, *cadD* was upregulated (Fig. 4B to D). Each of these transcriptional responses indicates metalation of the corresponding metallosensor (Fig. 4B to D), but whether by the cognate metal or by Cu cannot be distinguished. These observations were further corroborated by results from genome-wide RNA sequencing (RNA-seq) analyses. Multiple AdcR- and MtsR-controlled genes were negatively regulated, while both the CadC-controlled genes were positively regulated in response to 5 μM added Cu (Table 1; see Data Set S1). Interestingly, no clear effect on *gczA* or *czcD* expression was detected, suggesting that the metalation status of GczA, a TetR-family Zn-sensing derepressor (46), is not altered by Cu treatment.

**TABLE 1:** Cu treatment leads to a misregulation of metal homeostasis^*a*^

Gene	MGAS5005 gene product annotation	Log_2_FC(+Cu/–Cu)	*P*_adj_
CopY regulated			
*copZ*	Copper chaperone	4.41	0.0000
*copA*	Copper-exporting ATPase	4.39	0.0000
*copY*	CopAB ATPase metal-fist type repressor	4.49	0.0000
			
CadC regulated			
*cadD*	Cadmium resistance protein	3.15	0.0000
*cadC*	Cadmium efflux system accessory protein	4.00	0.0000
			
AdcR regulated			
*phtY*	Internalin protein	−1.80	0.0000
*rpsN*	30S ribosomal protein S14	−1.72	0.0000
*phtD*	Histidine triad protein	−2.51	0.0000
*adcAII*	Laminin-binding protein	−2.37	0.0000
*adhE*	Acetaldehyde-CoA/alcohol dehydrogenase	−3.32	0.0000
			
MtsR regulated			
*fhuG*	Ferrichrome transporter permease	−1.99	0.0000
*fhuB*	Ferrichrome transporter permease	−1.81	0.0000
*fhuD*	Ferrichrome-binding protein	−1.91	0.0000
*fhuA*	Ferrichrome ABC transporter ATP-binding protein	−1.75	0.0000
*nrdI.2*	Ribonucleotide reductase stimulatory protein	−1.57	0.0006
*nrdE.2*	Ribonucleotide-diphosphate reductase subunit alpha	−1.64	0.0001
*hupY*	Cell surface protein	−2.29	0.0000
*hupZ*	Hypothetical protein M5005_Spy_0652	−2.25	0.0000
*siaD*	ABC transporter ATP-binding protein	−2.34	0.0000
*siaC*	Ferrichrome ABC transporter ATP-binding protein	−2.71	0.0000
*siaB*	Ferrichrome transporter permease	−2.63	0.0000
*siaA*	Ferrichrome-binding protein	−2.28	0.0000
*shp*	Heme-binding protein	−1.80	0.0000
*shr*	Fe^3+^-siderophore transporter	−1.94	0.0000
			
GczA regulated			
*gczA*	TetR family transcriptional regulator	0.03	0.9378
*czcD*	Cobalt-zinc-cadmium resistance protein	0.46	0.2807
			
GSH import			
*gshT*	Amino acid ABC transporter substrate-binding protein	0.90	0.1098
*tcyB*	Amino acid ABC transporter permease	0.83	0.0409
*tcyC*	ABC transporter substrate-binding protein	0.87	0.0443

a GAS Δ*copA* mutant strain was cultured with or without 5 μM added Cu for *t* = 5 h (*n* = 3). Total RNA was extracted, rRNA was depleted, and cDNA was generated and finally sequenced by Illumina. Differential gene expression was determined using DeSeq2 and is presented as the fold change (FC) in gene expression in the Cu-treated cultures relative to that in the untreated control. Only genes of interest are listed. These are genes regulated by metal-sensing transcriptional regulators CopY, CadC (45), AdcR (43), MtsR (44), and GczA (46), as well as those that encode components of the putative GSH uptake system (50). A complete list of differentially regulated genes is provided in Data Set S1.

10.1128/mBio.02804-20.1 DATA SET S1 List of genes that were upregulated (A) and downregulated (B) in response to Cu treatment. GAS Δ*copA* mutant strain was cultured with or without 5 μM added Cu for *t* = 5 h (*n* = 3). Total RNA was extracted, rRNA was depleted, and cDNA was generated and finally sequenced by Illumina. Differential gene expression was determined using DeSeq2 and is presented as the fold change (FC) in gene expression in Cu-treated cultures relative to that in the untreated control. We used arbitrary cutoffs of log_2_FC > 1.5 for upregulated genes and log_2_FC < 1.5 for downregulated genes. Download Data Set S1, XLSX file, 0.02 MB. Copyright © 2020 Stewart et al. 2020 Stewart et al. This content is distributed under the terms of the Creative Commons Attribution 4.0 International license.

Crucially, changes in the expression of *adcAII*, *siaA*, and *cadD* appeared only after ∼4 h of growth (Fig. 4B to D). These transcriptional changes were not accompanied by increases in total intracellular Zn, Mn, or Fe levels (see Fig. S4). Thus, the simplest model that accounts for the sudden metalation (or mismetalation) of multiple metallosensors, as well as GapA, is that excess Cu is released from an intracellular buffer, leading to mislocation of Cu to adventitious binding sites and/or redistribution of intracellular metals.

### The onset of the Cu stress phenotype coincides with depletion of GSH.

What comprises the intracellular buffer for excess Cu in GAS? This organism does not possess a homologue of the metallothionein MymT (17) or the Cu storage protein Csp (47). Instead, this buffer likely consists of a polydisperse mixture of cytoplasmic small molecules or metabolites (48). Noting that GAS is auxotrophic for most nutrients, including multiple amino acids, vitamins, nucleobases, and GSH, we hypothesized that: (i) one or more of these nutrients constitute the intracellular Cu buffer, either directly by coordinating Cu or indirectly by acting as a synthetic precursor to the buffer, and that (ii) these nutrients become exhausted from the extracellular medium during bacterial growth, leading to the observable effects of Cu stress.

The above hypothesis was tested using two complementary approaches and the results identified GSH as the key limiting nutrient. First, mass spectrometry was employed to measure consumption of nutrients from the growth medium. Several amino acids, the nucleobases adenine and uracil, as well as GSH (and/or its disulfide GSSG) were nearly or completely spent after ∼4 h of growth (see Fig. S5). Cys and its disulfide were below detection limits. Next, the culture medium was supplemented with each or a combination of the spent or undetected extracellular nutrients. Their ability to restore growth of Cu-treated Δ*copA* mutant cultures was subsequently examined. Only supplementation with GSH was strongly protective against Cu intoxication (see Fig. S6).

10.1128/mBio.02804-20.7 FIG S5 Time-dependent changes in the levels of extracellular growth medium components: amino acids (A), vitamins (B), nucleobases (C), and glutathione (D). GAS Δ*copA* mutant strain was cultured without added Cu (*n* = 4). The cultures were centrifuged (5,000 × *g*, 10 min) and filtered through a 0.22-μm PES membrane at the indicated time points. The filtrates (spent media) were subjected to analyses by LC-MS on a Sciex QTRAP 6500 at Durham Biosciences Proteomics Facility. The amount of each amino acid, vitamin, and nucleobase in these filtrates was normalized to the amounts present at *t* = 0 h. The amino acid Cys was below detection limit. Glutathione levels were measured separately by Gor/DTNB recycling assay (I. Rahman, A. Kode, and S. K. Biswas, Nat Protoc 1:3159–3165, 2006). Medium components that were nearly or completely spent at *t* = 4 h are highlighted in red. Download FIG S5, TIF file, 1.3 MB. Copyright © 2020 Stewart et al. 2020 Stewart et al. This content is distributed under the terms of the Creative Commons Attribution 4.0 International license.

10.1128/mBio.02804-20.8 FIG S6 Effects of medium supplements on bacterial growth. GAS Δ*copA* mutant strain was cultured with added Cu as indicated. The cultures were also supplemented with GSH (1 mM, *n* = 3) (A), combinations of select amino acids (1 mM each, *n* = 3, single-letter amino acid codes are shown) (B), or vitamins and nucleobases (to 5× the initial concentration each, *n* = 3) (C). The corresponding untreated (water) control cultures for each experiment are shown on the left. Download FIG S6, TIF file, 0.8 MB. Copyright © 2020 Stewart et al. 2020 Stewart et al. This content is distributed under the terms of the Creative Commons Attribution 4.0 International license.

**FIG 5:**
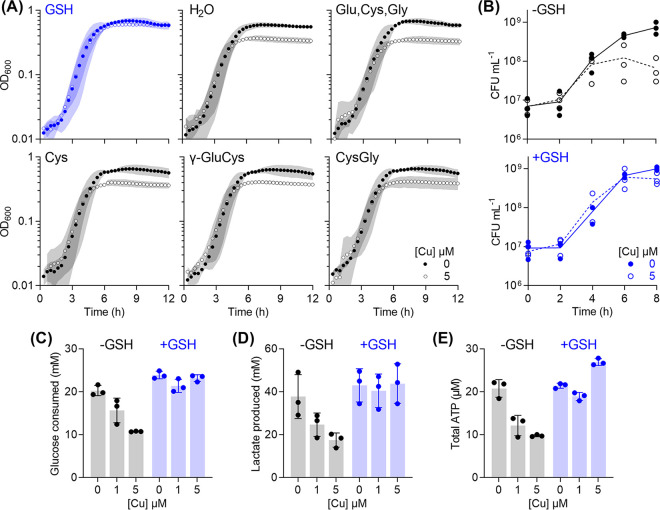
Protective effects of supplemental GSH. GAS Δ*copA* mutant strain was cultured with added Cu as indicated (*n* = 3) in the absence (black) or presence (blue) of 0.1 mM GSH. (A) Growth. Cu treatment did not affect GSH-supplemented cultures (*P* = 0.99). Growth curves in culture medium supplemented with water; a mixture of Glu, Cys, and Gly (0.1 mM each); Cys alone (0.1 mM); the dipeptide γ-GluCys (0.1 mM); or CysGly (0.1 mM) are also shown for comparison. (B) Plating efficiency. Cultures were plated out at the indicated time points and the numbers of CFU were enumerated. Cu treatment suppressed plating efficiency of GSH-deplete cultures (*P* = 0.0012) but not that of the GSH-supplemented cultures (*P* = 0.97). (C) Glucose consumption. Cultures were sampled at *t* = 8 h, and total amounts of glucose consumed from the extracellular growth media were determined. Cu treatment suppressed glucose consumption by GSH-deplete cultures (*P* = 0.0053 for 1 μM Cu, *P* < 0.0001 for 5 μM Cu) but not that by GSH-supplemented cultures (*P* = 0.12 for 1 μM Cu, *P* = 0.81 for 5 μM Cu). (D) Lactate production. Cultures were sampled at *t* = 8 h, and the amounts of lactate secreted to the extracellular growth media were determined. Cu treatment suppressed lactate production by GSH-deplete cultures (*P* = 0.11 for 1 μM Cu, *P* = 0.014 for 5 μM Cu) but not that by GSH-supplemented cultures (*P* = 0.91 for 1 μM Cu, *P* = 0.99 for 5 μM Cu). (E) Total ATP levels. Cultures were sampled at *t* = 8 h and total ATP levels were determined. Cu treatment suppressed ATP production by GSH-depleted cultures (*P* < 0.0001 each for 1 and 5 μM Cu) but not that by the GSH-supplemented cultures (*P* = 0.095 for 1 μM Cu, *P* = 0.0008 for 5 μM Cu). All statistical analyses were versus 0 μM Cu.

The GAS genome encodes neither the common pathway for GSH biosynthesis (GshAB) nor the bifunctional glutathione synthetase (GshF [49]). Instead, an uncharacterized homologue of the GSH-binding solute-binding protein GshT is present (M5005_Spy0270, 59% sequence identity, 74% sequence similarity with the characterized homologue from S. mutans) (50). GshT, in conjunction with the endogenous cystine importer TcyBC, likely allows GAS to import extracellular GSH (γ-Glu-Cys-Gly) into the cytoplasm (50). This system may also import γ-Glu-Cys or Cys-Gly (50), but addition of these dipeptides, or Cys alone, or a mixture of the amino acids Glu, Cys, and Gly did not improve growth of Cu-treated Δ*copA* mutant cultures (Fig. 5A). Altogether, these results suggest that: (i) the protective effect of GSH is unlikely to result from chelation of *extracellular* Cu ions by free thiols, (ii) extracellular GSH is depleted during growth of GAS, and (iii) this depletion is responsible for the observable Cu stress phenotypes. Consistent with propositions ii and iii, addition of GSH completely suppressed the effects of Cu treatment and restored plating efficiency, as well as glucose consumption, lactate secretion, and ATP production beyond the late exponential phase of growth (Fig. 5B to E).

### GSH contributes to buffering of excess intracellular Cu.

**FIG 6:**
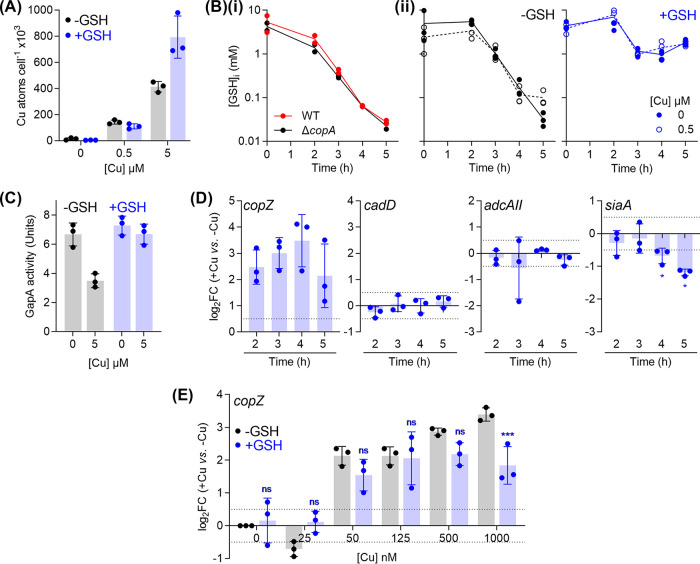
Buffering of excess intracellular Cu ions by GSH. GAS Δ*copA* mutant strain was cultured with added Cu as indicated, without (black) or with (blue) 0.1 mM added GSH. The following properties were subsequently measured. (A) Intracellular Cu levels. Cultures (*n* = 3) were harvested at *t* = 4 h, and total cellular levels of Cu were measured by ICP-MS. GSH supplementation did not affect intracellular Cu levels when cultures were treated with 0 (*P* = 1) or 0.5 μM Cu (*P* = 0.92), but it did lead to an increase in Cu levels when cultures were treated with 5 μM Cu (*P* < 0.0001). (B) Intracellular GSH concentrations. (i) Cultures (*n* = 2) were prepared without any added Cu or GSH and sampled at the indicated time points. Intracellular levels of GSH were measured in cell extracts. There was no clear difference between the time-dependent decrease in intracellular GSH levels of WT and Δ*copA* cultures. (ii) Cultures (*n* = 3) were sampled at the indicated time points and intracellular levels of GSH were measured in cell extracts. Cu treatment did not affect intracellular GSH levels, regardless of GSH supplementation (*P* = 0.95 for 0 mM GSH, *P* = 1.0 for 0.1 mM GSH). GSH supplementation improved intracellular GSH levels (*P* < 0.0001 for both 0 and 0.5 μM Cu), regardless of Cu treatment. (C) GapA activity. Cultures (*n* = 3) were harvested at *t* = 4 h. GapA activity was measured in cell extracts. Cu treatment suppressed GapA activity in GSH-depleted cultures (*P* = 0.0007) but not in GSH-supplemented cultures (*P* = 0.51). (D) Expression of metal homeostasis genes. GSH-supplemented cultures (*n* = 3) were sampled at the indicated time points. Levels of *copZ*, *cadD*, *adcAII*, and *siaA* transcripts in Cu-treated cultures were determined by qPCR and normalized to the corresponding untreated samples that were harvested at the same time points. Horizontal dotted lines represent the limit of the assay (log_2_FC = ±0.5). Cu treatment induced *copZ* expression (*P* = 0.023, 0.013, 0.026, and 0.093), but not *cadD* (*P* = 0.17, 0.71, 0.98, and 0.32), or *adcAII* (*P* = 0.39, 0.50, 0.03, and 0.16) at *t* = 2, 3, 4, and 5 h, respectively (versus log_2_FC = 0). Cu treatment continued to downregulate *siaA* expression (*P* = 0.63, 0.03, 0.04, and 0.03 for *t* = 2, 3, 4, and 5 h, respectively, versus log_2_FC = 0). (E) Cu-dependent expression of *copZ*. Cultures (*n* = 3) were sampled at *t* = 4 h. Levels of *copZ* transcripts in Cu-treated cultures were normalized to the corresponding untreated samples. Horizontal dotted lines represent the limit of the assay (log_2_FC = ±0.5). GSH supplementation did not affect *copZ* expression at low concentrations of added Cu (ns, *P* = 0.10, 0.14, 0.48, 1.0, and 0.31 for [Cu] = 0, 25, 50, 125, and 500 nM), but it did affect expression at 1000 nM added Cu (***, *P* = 0.0009).

To test that the protective effect of GSH is not linked to chelation of extracellular Cu ions and decreased uptake of Cu into the cytoplasm, total intracellular Cu levels were measured in Δ*copA* mutant cultures that were supplemented with Cu and/or GSH. As expected, GSH supplementation did not suppress total intracellular Cu levels when Δ*copA* cultures were challenged with low concentrations of added Cu (500 nM; Fig. 6A). Surprisingly, at high concentrations of added Cu (5 μM), GSH-replete cultures appeared to accumulate higher, rather than lower, intracellular Cu levels (Fig. 6A). Yet, these cultures did not display an observable Cu stress phenotype (Fig. 5). These findings are discussed below.

The time-dependent reduction in extracellular GSH levels (see Fig. S5D) was mirrored by a decrease in intracellular GSH (Fig. 6Bi). Both the wild-type and Δ*copA* mutant strains contained ∼4 mM intracellular GSH (and GSSG) at *t* = 0 h (Fig. 6Bi). This amount was likely already present in the inoculum, which was cultivated in the complex medium THY ([GSH]_THY_ ∼ 30 μM [51]). Intracellular GSH levels in both strains reduced to ∼0.1 mM at *t* = 4 h, regardless of Cu treatment (Fig. 6Bi and ii). This decrease occurred presumably as a consequence of bacterial growth and replication in a chemically defined medium with a limited GSH supply ([GSH]_CDM_ ∼ 0.5 μM; see Fig. S5D). The low amount of intracellular GSH coincided with the onset of the observable Cu stress phenotypes. It might also explain why cultures that grew to low OD_600_ values displayed no sign of Cu stress (see Fig. S2C and D); these cultures likely had not depleted their intracellular GSH supply.

Notably, Cu treatment did not transcriptionally induce the uptake of GSH. Levels of *gshT* transcripts remained largely unchanged, based on RNA-seq analyses of *ΔcopA* cells at the late-exponential phase of growth (Table 1). This result supports previous transcriptomic studies in several Gram-positive and Gram-negative bacteria, none of which identified GSH biosynthesis or uptake as a key transcriptional response to Cu treatment (15, 20, 22, 23).

Supplementation of the growth medium with GSH (0.1 mM) did not affect the intracellular levels of this thiol at the early stages of growth (*t* = 0 and 2 h; Fig. 6Bii). However, it did allow Δ*copA* cells to maintain intracellular concentrations of this tripeptide at ∼1 mM (one log unit higher than unsupplemented cells) beyond the late exponential growth phase, regardless of Cu treatment (Fig. 6Bii). As mentioned earlier, these GSH-treated cells were Cu-tolerant (Fig. 5). In fact, these cells accumulated more intracellular Cu compared with the GSH-untreated control (Fig. 6A). The simplest explanation for this finding is that the rise in intracellular GSH levels leads to an increased ability to buffer intracellular Cu. A more detailed examination of GSH-supplemented Δ*copA* cells confirmed that GapA was protected from inactivation by added Cu (Fig. 6C). In addition, the Cu-induced, time-dependent changes in *cadD* and *adcAII* expression were abolished (Fig. 6D), suggesting that CadC and AdcR did not become mismetalated. Some downregulation of *siaA* transcription was observed, albeit to a lesser magnitude compared with GSH-deplete cultures (Fig. 6D versus Fig. 4D). In general, these results support a model whereby GSH constitutes the major buffer for excess intracellular Cu in GAS and protects potential noncognate binding sites from becoming (mis)metalated by Cu.

Importantly, GSH supplementation did not affect expression of *copZ* at low concentrations of added Cu (0 to 500 nM; Fig. 6E). This observation further strengthens the proposal that GSH does not rescue the Δ*copA* mutant simply by chelating extracellular Cu ions. However, GSH treatment did partially suppress *copZ* expression in response to a high concentration of added Cu (1,000 nM; Fig. 6E). This observation indicates the relative buffering strengths of GSH and CopY, which are discussed below.

## DISCUSSION

### Role of GSH in buffering excess cytoplasmic Cu.

GSH has been proposed to bind Cu by assembling a stable, tetranuclear Cu_4_GS_6_ cluster (52). In such a model, when present at low millimolar concentrations (e.g., ∼4 mM in GAS at *t* = 0 h; Fig. 6A), GSH would bind Cu with an apparent affinity of *K_D_* = 10^−16.7^ M and thus would impose a threshold of Cu availability at 10^−16.7^ M (see Fig. S7A). This threshold is above the range of Cu availability set by most bacterial Cu sensors (see Fig. S7B) (53–55). Therefore, GSH contributes to Cu buffering only when the transcriptionally responsive Cu homeostasis system is impaired (e.g., in a Δ*copA* mutant [25, 26]) or overwhelmed (e.g., when intracellular Cu levels rise above the responsive range of the Cu sensors).

10.1128/mBio.02804-20.9 FIG S7 Buffering of intracellular Cu by GSH. (A) Time-dependent and GSH-dependent changes in the ability of GSH to buffer intracellular Cu. The threshold of Cu availability imposed by GSH (plotted as the log[Cu]) was estimated using equation 4 in M. T. Morgan et al. (J Biol Chem 292:21558–21567, 2017). Intracellular pH was assumed to be 7.2. Intracellular GSH concentrations at each time point were measured in Fig. 6A ([GSH] = 4.1, 3.3, 0.76, 0.11, and 0.03 mM at *t* = 0, 2, 3, 4, and 5 h, respectively). This estimate is valid only in the region shaded in grey, i.e., where GSH is present in a huge excess ([GSH]/[Cu] ≫ 20) (M. T. Morgan, L. A. H. Nguyen, H. L. Hancock, and C. J. Fahrni, J Biol Chem 292:21558–21567, 2017). (B) Comparison of GSH with characterized bacterial Cu sensors. The thresholds of Cu availability imposed by CsoR from B. subtilis (Z. Ma, D. M. Cowart, R. A. Scott, and D. P. Giedroc, Biochemistry 48:3325–3334, 2009), CopY from S. pneumoniae (H. Glauninger, Y. Zhang, K. A. Higgins, A. D. Jacobs, et al., Chem Sci 9:105–118, 2018), and CueR from *S.* Typhimurium (D. Osman, M. A. Martini, A. W. Foster, J. Chen, et al., Nat Chem Biol 15:241–249, 2019) were estimated using the calculator developed by D. Osman et al. (Nat Chem Biol 15:241–249, 2019) (see Data Set S2C). The midpoint threshold imposed by 4 mM GSH is shown as straight dashed line. (C) Effect of Cu affinity on the CopY sensor. The thresholds of Cu availability imposed by CopY were estimated using the using published Cu binding affinity of log *K*_Cu_ = 16.6 (solid pink curve) (H. Glauninger, Y. Zhang, K. A. Higgins, A. D. Jacobs, et al., Chem Sci 9:105–118, 2018) and a 10× tighter affinity of log *K*_Cu_ = 17.6 (dashed pink curve). The midpoint threshold imposed by 4 mM GSH is shown as straight dashed line. (D) Alignment of CopY protein sequences. The two Cys-X-Cys motifs are boxed for clarity. NCBI GenBank accession numbers are shown in brackets. Download FIG S7, TIF file, 1.8 MB. Copyright © 2020 Stewart et al. 2020 Stewart et al. This content is distributed under the terms of the Creative Commons Attribution 4.0 International license.

10.1128/mBio.02804-20.2 DATA SET S2 (A) Detailed composition of growth medium used in this study. (B) List of primers, strains, and plasmids used in this study. (C) Values used to generate the model in Fig. S7B. Download Data Set S2, XLSX file, 0.02 MB. Copyright © 2020 Stewart et al. 2020 Stewart et al. This content is distributed under the terms of the Creative Commons Attribution 4.0 International license.

Figure 6E shows that supplementation with GSH had little impact on metalation of CopY (and thus expression of *copZ*) when the amounts of added Cu were low. However, GSH appeared to dampen CopY response at higher concentrations of added Cu, indicating that this thiol competes with CopY for binding Cu when intracellular Cu levels are high. Hence, the thresholds of intracellular Cu availability set by GSH and CopY may overlap, at least partially, with GSH being the weaker buffer (52, 55, 56). The thermodynamic model in Fig. S7B is compatible with these experimental data, but it will need refinement. This model was estimated using known parameters (Cu affinity, DNA affinity, and number of DNA targets) for CopY from S. pneumoniae (CopY_Spn_) (55), but CopY_Spn_ differs from CopY_GAS_ in several key aspects. CopY_Spn_ lacks one of the two Cys-X-Cys motifs found in other CopY homologues such as CopY_GAS_ and CopY from *E. hirae* (CopY_Eh_) (see Fig. S7D). CopY_Spn_ binds two Cu atoms per dimer in a solvent-exposed center while CopY_Eh_ binds four Cu atoms per functional dimer and assembles a solvent-occluded center (55, 57). In addition, two *cop* boxes are present in S. pneumoniae (58), while only one is found in GAS. How these differences shift the threshold model will need to be examined using careful *in vitro* studies with purified proteins and DNA. In the simplest scenario, an increase in the stability (affinity) of the bound Cu atoms in CopY, which may occur as a consequence of coordination by extra Cys ligands, would lower the threshold of Cu availability set by CopY (see Fig. S7C) and thus better fit our experimental data.

Depletion of intracellular GSH to 0.1 mM at the late exponential phase of growth would weaken its buffering capacity by at least 2 log units (see Fig. S7A). Figure 4 shows that Cu is then able to metalate nonspecific binding sites in non-cognate metallosensors or metalloenzymes. These results further suggest that AdcR, CadC, and MtsR can allosterically respond to Cu and differentially regulate expression of their target genes *in vivo*. Precisely how this occurs will need to be confirmed with purified proteins and DNA *in vitro.* Cu-responsive regulation of genes under the control of non-cognate metallosensors has indeed been reported both *in vivo* and *in vitro*, although not for the families of regulators described here (15, 28, 59–61).

Not all bacteria use GSH as the major cytoplasmic thiol. Some bacilli, such as B. subtilis and S. aureus, produce the glycoside bacillithiol (BSH) instead. The affinity of BSH to Cu is at least 2 orders of magnitudes tighter than that of GSH (56, 62). Hence, BSH likely imposes a lower limit on cytoplasmic Cu availability than does GSH, but it is worth noting that its intracellular level is ∼30 times lower than that of GSH (63). Importantly, the relative order with the endogenous Cu sensor CsoR still holds, with BSH binding Cu at least 3 log units more weakly than does CsoR (54). Indeed, this thiol is also thought to contribute to Cu homeostasis by buffering excess Cu. Deletion of the B. subtilis *bshC* gene for BSH biosynthesis led to a slight increase in *copZ* expression in response to added Cu. This result mirrors the finding in Fig. 6E and suggests that the Cu sensor CsoR is more readily metalated by Cu in the absence of the major buffering thiol (64). It is also notable that the identification of GapA as a major reservoir of excess Cu ions in the cytoplasm was in a strain of S. aureus that does not synthesize BSH (39).

**FIG 7:**
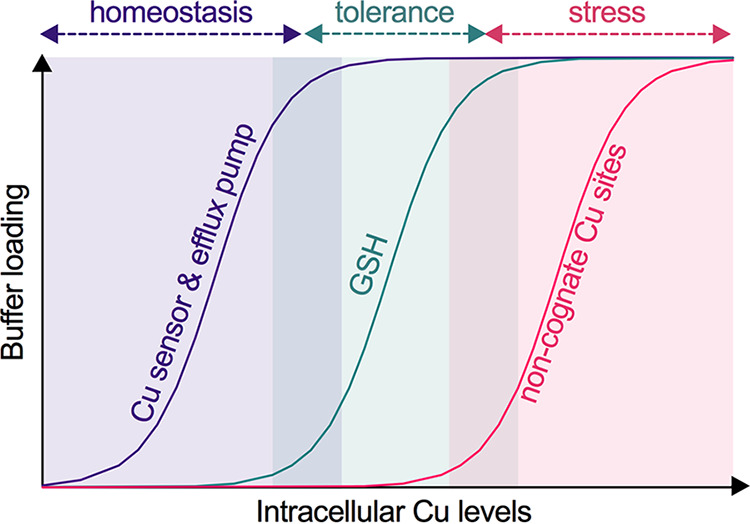
Threshold model for bacterial Cu homeostasis, tolerance, and stress. As Cu levels in the cytoplasm increase, this metal ion binds to the allosteric site of the Cu-sensing transcriptional regulator, which subsequently induces expression of the Cu efflux pump. Together, the Cu sensor and efflux pump impose a low limit of Cu availability and maintain Cu homeostasis. A further rise in cytoplasmic Cu levels saturates the Cu homeostasis system and begins to fill binding sites in GSH. Since there are no observable defects in bacterial metabolism or growth at this stage, GSH can be considered to confer Cu tolerance. GSH depletion or a further increase in cytoplasmic Cu levels saturates this tolerance capacity. Cu now binds to noncognate metal-binding sites, leading to inhibition of bacterial metabolism and growth. These conditions are considered as Cu stress.

In summary, this study provides a new line of evidence that Cu handling in the bacterial cytoplasm, when formulated using the threshold model, comprises two components (Fig. 7). The transcriptionally responsive component, which includes the Cu sensor, Cu efflux pump, and additional Cu-binding metallochaperones, functions in housekeeping or homeostasis and sets a low limit of Cu availability in the cytoplasm. Rising Cu levels can saturate this homeostasis system and sudden Cu shock can overwhelm it, but the transcriptionally unresponsive component, in this case GSH, buffers the excess Cu and confers additional Cu tolerance. This second system acts as the final layer of protection before cells experience widespread mismetalation and, therefore, Cu stress (Fig. 7). This additional buffering essentially extends the range of cytoplasmic Cu availability that can be tolerated by bacteria, allows bacteria to maintain key cellular functions, and thus prevents an abrupt transition from Cu homeostasis to Cu stress upon exposure to an excess of this metal ion. While this study focused exclusively on a Gram-positive bacterial organism, this concept is likely to apply to other bacterial systems and mammalian models (65).

### Role of GSH in buffering bacterial Cu during host-pathogen interactions.

This study was conducted originally to examine the role of the Cop Cu homeostasis system in GAS pathogenesis. Although GAS occupied a Cu-rich environment in mice (Fig. 1A), inactivation of the *copA* gene did not lead to a reduction in GAS virulence (Fig. 1C). Our *in vitro* investigations now suggest that GAS may withstand host-imposed increases in Cu levels, as long as it has access to a source of GSH *in vivo*. Indeed, GSH was detected in the skin ulcers of infected mice, but interestingly, the amount was ∼25-fold less compared to skin from healthy mice or healthy skin from infected mice (see Fig. S8). Whether this depletion of GSH is a feature of the general host immune response, a consequence of inflammation and/or host tissue necrosis, or a consequence of GAS metabolism is not known. Nevertheless, the virulence of the Δ*copA* mutant implies that the level of host GSH, albeit reduced, can support Cu buffering inside the GAS cytoplasm. Alternatively, the level of host Cu (Fig. 1A) may not be sufficient to overwhelm the Cop homeostasis system, since the *copYAZ* operon was only slightly upregulated in bacteria isolated from mouse ulcers (average log_2_FC = 1.13 versus THY) (34).

10.1128/mBio.02804-20.10 FIG S8 Amounts of host GSH in the ulcerative lesions. Mice were infected subcutaneously with GAS wild-type strain. After 3 days, the skin of uninfected mice (naive), skin lesions from infected mice, and control skin adjacent to the lesions of infected mice were excised (*n* = 3 each). Total GSH levels were measured and normalized to the weight of the tissue sample. There was a clear reduction in GSH levels in the lesions from infected mice compared to healthy skin from uninfected mice (*P* = 0.0059) and adjacent healthy skin from infected mice (*P* = 0.0059). Download FIG S8, TIF file, 0.1 MB. Copyright © 2020 Stewart et al. 2020 Stewart et al. This content is distributed under the terms of the Creative Commons Attribution 4.0 International license.

### Link between the failure to buffer Cu and redox stress.

Under our experimental conditions, untreated Δ*copA* cells contained 17,000 to 23,000 Cu atoms when sampled at *t* = 3 h (before the onset of Cu stress). Cu treatment increased this number to 78,000 to 330,000 atoms (see Fig. S4). The intracellular GSH concentrations at the same time point ([GSH]_i_ = 0.76 mM; Fig. 6B) would translate to ∼500,000 molecules of GSH, which are clearly insufficient to buffer all of the intracellular Cu ions. However, there was no observable Cu stress phenotype at this time point, suggesting that the excess Cu is also bound to other cytoplasmic component(s). These components may include CopZ and/or the novel, uncharacterized protein CopX (see Fig. S1A). This idea will be the focus of future studies.

Finally, the GSH/GSSG couple is the major redox buffer of the cell. Assuming that the GSH/GSSG ratio remains unchanged, depletion of intracellular GSH in GAS from ∼4 to ∼0.1 mM would raise the cytoplasmic redox midpotential by ∼46 mV. This relatively more oxidizing environment, when combined with a lack of Cu buffering, may promote the Cu-catalyzed generation of reactive oxygen species (66) or the formation of disulfides (67). Yet, our RNA-seq results do not suggest widespread oxidative stress (see Data Set S1). In E. coli, deletion of *gshA* did not accelerate DNA damage in Cu-replete cells, even in the presence of added H_2_O_2_ (68). Similarly, proteomic analyses of a non-BSH-producing strain of S. aureus indicated that Cu treatment does not induce a strong oxidative stress response in this organism (39).

Regardless of the relative importance of mismetalation *versus* redox stress, our work demonstrates that excess Cu is not bacteriotoxic as long as cytoplasmic GSH is abundant and thus able to buffer the excess of this metal ion (Fig. 7). In GAS, a GSH auxotroph, this intracellular buffer is dynamic; its levels change during bacterial growth and/or in response to extracellular GSH availability. Future studies should take these effects into account when examining the impact of Cu treatment on bacterial cultures. Had our work not identified the 4-h time point as metabolically relevant, sampling cultures 1 h earlier would have led to a different conclusion.

## MATERIALS AND METHODS

### Data presentation and statistical analyses.

We follow recent recommendations regarding transparency in data representation (69, 70). Except for growth curves, individual data points from independent experiments are plotted, with shaded columns representing the means and error bars representing standard deviations. Growth curves show the means of independent experiments, with shaded regions representing standard deviations. The number of independent experiments is stated clearly in each figure legend. Statistical analyses have been performed on all numerical data, but notations of statistical significance are displayed on plots only if they aid in rapid, visual interpretation. Otherwise, *P* values for key comparisons are stated in the figure legends. Unless otherwise stated, statistical tests used two-way analysis of variance using the statistical package in GraphPad Prism 8.0. All analyses were corrected for multiple comparisons.

### Ethics statement.

Animal experiments were conducted according to the Guidelines for the Care and Use of Laboratory Animals (National Health and Medical Research Council, Australia) and were approved by the University of Queensland Animal Ethics Committee (Australia). Human blood donation for use in neutrophil killing studies was conducted in accordance with the National Statement on Ethical Conduct in Human Research and in compliance with the regulations governing experimentation on humans, and was approved by the University of Queensland Medical Research Ethics Committee (Australia).

### Reagents.

All reagents were of analytical grade and obtained from Sigma or Melford Chemicals unless otherwise indicated. γ-Glu-Cys and Cys-Gly were from BACHEM Peptides (Germany). The sulfate and chloride salts of copper were used interchangeably. All reagents were prepared in deionized water.

### Strains and culture conditions.

GAS M1T1 5448 strains were propagated from frozen glycerol stocks onto solid THY medium without any antibiotics. Unless otherwise indicated, liquid cultures were prepared in a chemically defined medium containing glucose as the carbon source (CDM-glucose; see Data Set S2A). This medium routinely contained 53 nM basal Cu, 155 nM Zn, 66 nM Fe, 9 nM Mn, 29 nM Co, and 23 nM Ni, as determined by inductively coupled plasma mass spectrometry (ICP-MS). All solid and liquid growth media contained catalase (50 μg/ml).

### Construction of mutants.

Nonpolar GAS mutant strains were constructed by allelic exchange following standard protocols (71). Primers and plasmids used in this study are listed in Data Set S2B. All constructs and genetically altered strains were confirmed by PCR and Sanger sequencing.

### Mice virulence assays.

Transgenic, human plasminogenized *AlbPLG1* mice heterozygous for the human transgene were backcrossed greater than *n* = 6 with C57BL/J6 mice as described previously (72). GAS was prepared to obtain the target dose in the 10^7^ CFU range (WT, 1.8 × 10^7^; Δ*copA*, 1.5 × 10^7^) immediately prior to injection. Mice were subcutaneously infected (*n* = 10), and virulence was determined by observing survival for 10 days post-infection. Metal levels in mouse blood and skin were measured by ICP-MS as described previously (73).

To assess GSH levels at the site of infection, mouse skin and infected lesions were excised 3 days post-infection, washed with phosphate-buffered saline (PBS), resuspended in 1 ml of PBS, homogenized in Lysing Matrix F tubes using a FastPrep 24G instrument (MP Biomedicals; 4°C, speed 6, 40 s, 2 cycles), and centrifuged (10,000 × *g*, 5 min, 4°C). Total GSH was measured from the supernatant using the GSH-Glo kit (Promega) according to the manufacturer’s instructions, with the modification of mixing undiluted samples 1:1 with 2 mM Tris(2-carboxyethyl)phosphine (TCEP) immediately prior to use.

### Neutrophil-killing assays.

Survival of GAS after incubation with human neutrophils *ex vivo* was assayed at a multiplicity of infection of 10:1 as previously described (72).

### Bacterial growth.

Growth was assessed at 37°C in flat-bottomed 96-well plates using an automated microplate shaker and reader. Each well contained 200 μl of culture. Each plate was sealed with a gas permeable, optically clear membrane (Diversified Biotech). OD_600_ values were measured every 20 min for 12 h. The plates were shaken at 200 rpm for 1 min in the double orbital mode immediately before each reading. OD_600_ values were not corrected for path length (ca. 0.58 cm for a 200-μl culture).

### Plating efficiency.

GAS was cultured in 96-well plates as described earlier for growth analysis, sampled at the indicated time points, vortexed for 30 s, diluted serially in PBS, and plated onto solid THY medium without any antibiotics. Colonies were enumerated after overnight incubation at 37°C.

### ATP levels.

GAS was cultured in 96-well plates as described earlier for growth analysis and sampled at the indicated time points. The amount of total ATP in each sample was determined immediately using the BacTiter-Glo kit (Promega).

### Intracellular metal content.

GAS was cultured in 10 to 500 ml of CDM-glucose as required (larger volumes were required to obtain enough biomass at earlier time points). At the desired time points, an aliquot was collected for the measurement of OD_600_ or plating efficiency. The remaining cultures were harvested (5,000 × *g*, 4°C, 10 min) and then washed once with PBS containing EDTA (1 mM) and twice with ice-cold PBS. The final pellet was dissolved in concentrated nitric acid (150 μl, 80°C, 1 h) and diluted to 10 ml with deionized water. Total metal levels were determined by ICP-MS. The results were normalized to OD_600_ values or plating efficiency as indicated in the figure legends.

### Fermentation end products.

GAS was cultured in 96-well plates as described earlier for growth analysis. At the desired time points, samples were centrifuged (5,000 × *g*, 4°C, 10 min) and the supernatants were frozen at –20°C until further use. Concentrations of pyruvate, lactate, acetate, and ethanol in the spent culture media were determined by using K-PYRUV, K-LATE, K-ACET, and K-ETOH kits (Megazyme), respectively. Concentrations of glucose were determined using the GAGO20 kit (Sigma).

### Enzyme activity.

GAS was cultured in 40 to 250 ml of CDM-glucose as required (larger volumes were required to obtain enough biomass at earlier time points). At the desired time points, bacteria were harvested (5,000 × *g*, 4°C, 10 min), washed once with PBS, and frozen at –20°C until further use. Bacterial pellets were resuspended in a buffer containing sodium phosphate (100 mM) and triethanolamine (80 mM) at pH 7.4, transferred to a tube containing Lysing Matrix B (MP Biomedicals), and lysed in a FastPrep 24G instrument (MP Biomedicals, 10 m/s, 20 s, 2 cycles). Cell debris were removed by centrifugation (20,000 × *g*, 1 min). The cell-free lysate supernatant was kept on ice and used immediately.

To determine GapA activity, the reaction mixture contained NAD^+^ (4 mM), dl*-*glyceraldehyde-3-phosphate (G3P; 0.3 mg/ml), sodium phosphate (100 mM), dithiothreitol (1 mM), and triethanolamine (80 mM) at pH 7.4. GapN activity was determined as described above for GapA but using NADP^+^ (4 mM) instead of NAD^+^ as the electron acceptor. To measure the activity of Ldh, the reaction mixture contained NADH (4 mM), pyruvate (10 mM), and fructose-1,6-bisphosphate (1 mM) in PBS at pH 7.4. For all three enzymes, each reaction (100 μl) was initiated by addition of cell extracts (10 μl). Absorbance values at 340 nm were monitored for up to 10 min at 37°C. The initial rates of reaction were normalized to total protein content as determined using the QuantiPro BCA assay kit (Sigma). Control reactions without any substrate (G3P for GapA and GapN, pyruvate for Ldh) were always performed in parallel.

One unit of activity was defined as follows: 1,000 nmol NAD^+^ oxidized min^−1 ^mg protein^−1^ for GapA, 100 nmol NADP^+^ oxidized min^−1 ^mg protein^−1^ for GapN, and 1,000 nmol NADH reduced min^−1 ^mg protein^−1^ for Ldh.

### GSH levels.

GAS was cultured in 10 to 150 ml of CDM-glucose as required (larger volumes were required to obtain enough biomass at earlier time points). At the desired time points, an aliquot was plated for bacterial counting. The remaining cultures were harvested (5,000 × *g*, 4°C, 10 min), washed twice with PBS, resuspended in 5-sulfosalycylic acid (5 wt/vol %), transferred to a tube containing Lysing Matrix B, and frozen at –20°C until further use. Bacteria were lysed in a bead beater (10 m/s, 30 s, 2 cycles). Cell debris were removed by centrifugation (20,000 × *g*, 1 min). Total GSH (and GSSG) levels in lysate supernatants were determined immediately using the Gor-DTNB recycling method (74) and normalized to total bacterial counts.

### RNA extraction.

GAS was cultured in 2 to 200 ml of CDM-glucose as required (larger volumes were required to obtain enough biomass at earlier time points). At the desired time points, cultures were centrifuged (3,000 × *g*, 4°C, 5 min). Bacterial pellets were resuspended immediately in 1 ml of RNAPro solution (MP Biomedicals) and stored at –80°C until further use. Bacteria were lysed in Lysing Matrix B, and total RNA was extracted according to the manufacturer’s protocol (MP Biomedicals). RNA extracts were treated with RNase-Free DNase I enzyme (New England Biolabs). Complete removal of gDNA was confirmed by PCR using gapA-check-F/R primers (see Data Set S2B). gDNA-free RNA was purified by using an RNeasy minikit (Qiagen) and visualized on an agarose gel.

### qPCR analyses.

cDNA was generated from 1 μg of RNA using the SuperScript IV first-strand synthesis system (Invitrogen). qPCR was performed in 10- or 20-μl reactions using 2 or 5 ng of cDNA as the template and 0.4 μM concentrations of the appropriate primer pairs (see Data Set 2B). Each sample was analyzed in technical duplicates. Amplicons were detected with PowerUP SYBR green (Invitrogen) in a QuantStudio 6 Flex real-time PCR system (Applied Biosystems) or a CFXConnect Real-Time PCR Instrument (Bio-Rad Laboratories). *C_q_* values were calculated using LinRegPCR after correcting for amplicon efficiency. *C_q_* values of technical duplicates were typically within ±0.25 of each other.*holB* and *tufA*, which encode DNA polymerase III and elongation factor Tu, respectively, were used as reference genes (see Data Set S2B). Their transcription levels remained constant in all of the experimental conditions tested here. *holB* was used as the reference gene in all the data presented here because its *C_q_* values were closer to the dynamic ranges of *cop* genes, *adcAII*, *cadD*, and *siaA*, but the results were identical with when *tufA* was used as the reference.

### RNA-seq analyses.

GAS Δ*copA* mutant strain was cultured in the presence of 0 or 5 μM added Cu for *t* = 5 h (*n = *3), and RNA was extracted from each culture as described earlier. RNA-seq was performed from Ribo-zero (rRNA-depleted) triplicate samples on a single Illumina HiSeq 2500 lane using v4 chemistry from 75-bp paired-end reads. Reads were mapped to the 5448 (M1) GAS reference genome (GenBank accession number CP008776.1) with BWA MEM (v0.7.16). Relative read counts (per gene) and differential gene expression was determined using DESeq2 (v1.26.0) (75) in R. Genes with fewer than 10 reads across all conditions and samples were removed. *P* values were calculated using Wald test and adjusted for multiple testing using the Benjamini-Hochberg method for controlling the false discovery rate. Illumina read data were deposited in the European Nucleotide Archive Sequence Read Archive under accession numbers ERS1996831, ERS1996835, and ERS1996839.
